# Calciprotein crystallization time (T_50_) and its association with surrogate cardiovascular disease risk markers in individuals with type 2 diabetes mellitus: the cross-sectional EARLY-HFpEF study

**DOI:** 10.1186/s12933-025-03016-9

**Published:** 2025-12-10

**Authors:** R. Meer, A. G. Hoek, E. Dal Canto, T. Doesburg, A. Pasch, M. G. Vervloet, P. A. de Jong, P. J. M. Elders, J. W. J. Beulens

**Affiliations:** 1 Department of Epidemiology & Data Science, Amsterdam UMC, Amsterdam, The Netherlands; 2 Amsterdam Cardiovascular Sciences Research Institute, Amsterdam, The Netherlands; 3 Laboratory of Experimental Cardiology, University Medical Centre Utrecht, Utrecht, The Netherlands; 4 Department of Radiology, Dijklander Ziekenhuis, Hoorn, The Netherlands; 5 Calciscon AG, Biel, Switzerland; 6 Institute for Physiology and Pathophysiology, Johannes Kepler University Linz, Linz, Austria; 7 Department of Nephrology, Radboud University Medical Centre, Nijmegen, The Netherlands; 8 Department of Radiology, University Medical Centre Utrecht, Utrecht, The Netherlands; 9 Department of General Practice, Amsterdam UMC, Amsterdam, The Netherlands; 10 Amsterdam Public Health Research Institute, Amsterdam, The Netherlands; 11 Julius Centre for Health Sciences and Primary Care, University Medical Centre Utrecht, Utrecht, The Netherlands

**Keywords:** Epidemiology, T_50_, Serum calcification propensity time, Calciprotein crystallization time, Heart failure with preserved ejection fraction, Ankle-brachial index, Peripheral artery disease, Arterial stiffness, Arterial calcification, Diabetes mellitus type 2

## Abstract

**Background:**

Heart failure and peripheral artery disease (PAD) are the two most common cardiovascular diseases (CVD) in individuals with type 2 diabetes mellitus (T2DM). The T_50_ calciprotein crystallization test measures the transformation of calciprotein particles type 1 (CPP1) into CPP2 in vitro and has been introduced as a low-cost biomarker for arterial calcification and CVD risk. We aimed to investigate the association between T_50_ and (1) heart failure with preserved ejection fraction (HFpEF), (2) ankle-brachial index (ABI) as measure of PAD, (3) pulse wave velocity (PWV) as measure of central arterial stiffness and (4) arterial calcification in individuals with T2DM.

**Methods:**

Cross-sectional data was used of 771 individuals with T2DM (64% men, 67 [63–71] years). T_50_ was measured using nephelometry on non-fasting serum samples. Presence of HFpEF was assessed with echocardiography based on current guidelines. ABI was categorized as ≤ 0.9 (PAD), 0.9–1.4 (normal) and ≥ 1.4 (high). Central arterial stiffness was measured using carotid-femoral PWV. Lower-extremity and coronary calcification were measured using computed tomography and quantified using Agatston scores categorized into zero (reference category) and tertiles > 0. Multivariable-adjusted Poisson, multinomial and linear regression analyses were used to study the associations with aforementioned surrogate CVD risk markers.

**Results:**

Mean T_50_ was 355 ± 55 min. HFpEF and PAD were present in 36.6% and 5.8% of the cohort, respectively. Mean cfPWV was 12.9 ± 2.5 m/s. Median calcification scores in the coronary arteries and lower-extremities were 315 [40–1246] and 791 [64–3820] Agatston units, respectively. Every 60-min decrease in T_50_, indicating higher calcification risk, was associated with increased coronary arterial calcification (e.g. highest tertile OR = 1.63 [1.15–2.30], *p* = 0.006), but not with lower-extremity arterial calcification (e.g. highest tertile OR = 1.28 [0.96–1.69], *p* = 0.088). Moreover, T_50_ ≤ 330 min versus T_50_ ≥ 390 min was associated with PAD (OR = 3.04 [1.03–8.94], *p* = 0.044). Finally, every 60-min decrease in T_50_ was not associated with neither HFpEF (RR = 1.02 [0.90–1.17], *p* = 0.736) nor cfPWV (β =  − 0.08 [ − 0.26–0.10], *p* = 0.398).

**Conclusion:**

Low T_50_ was associated with increased risks of coronary arterial calcification and PAD (measured by ABI ≤ 0.9) in individuals with T2DM, but not with HFpEF, central arterial stiffness and lower-extremity arterial calcification. Further research is warranted to evaluate the additive value of T_50_ in CVD risk stratification in clinical care.

**Supplementary Information:**

The online version contains supplementary material available at 10.1186/s12933-025-03016-9.

## Research insights

**What is currently known about this topic?**

Cardiovascular disease (CVD) is common in individuals with type 2 diabetes mellitus (T2DM).

The T_50_ test was introduced as a potential marker for CVD risk, including arterial calcification.

Low T_50_ indicates fast precipitation of calcium phosphate in serum.

**What is the key research question?**

What is the association between T_50_ and (1) heart failure with preserved ejection fraction (HFpEF), (2) ankle-brachial index (ABI) as measure of peripheral artery disease (PAD), (3) pulse wave velocity (PWV) as measure of central arterial stiffness and (4) arterial calcification in individuals with T2DM?

**What is new?**

The T_50_ may aid in CVD risk stratification in individuals with T2DM in primary care. 

Low T_50_ was associated with higher coronary artery calcification.

Low T_50_ was associated with ABI ≤ 0.9 (PAD), but not ABI ≥ 1.4 and PWV.

Low T_50_ was not associated with HFpEF.

**How might this study influence clinical practice?**

The T_50_ may aid in CVD risk stratification in individuals with T2DM in primary care.

### Background

Cardiovascular disease (CVD) is the principal cause of death in individuals with type 2 diabetes mellitus (T2DM) [1]. This is in part attributable to a high arterial calcification burden, conferring a three to four-fold increased risk of CVD [2, 3]. Moreover, heart failure and peripheral artery disease (PAD) are the two most prevalent forms of CVD in T2DM, accounting for an estimated 14 and 16% of incident CVD in these individuals, respectively [4]. The majority of incident heart failure cases (80%) consists of heart failure with preserved ejection fraction (HFpEF) in this population [5].

Various imaging modalities such as computed tomography (CT) and angiography are used in clinical practice to quantify arterial calcifications and estimate CVD risk. However, these modalities are not ideal for large epidemiological studies or frequent use in clinical care due to their high costs and exposure to radiation. As such, there is need for a low-cost and safe marker for CVD risk stratification, especially in a low-risk or low-income populations in which imaging is considered too expensive.

**Fig. 1:**
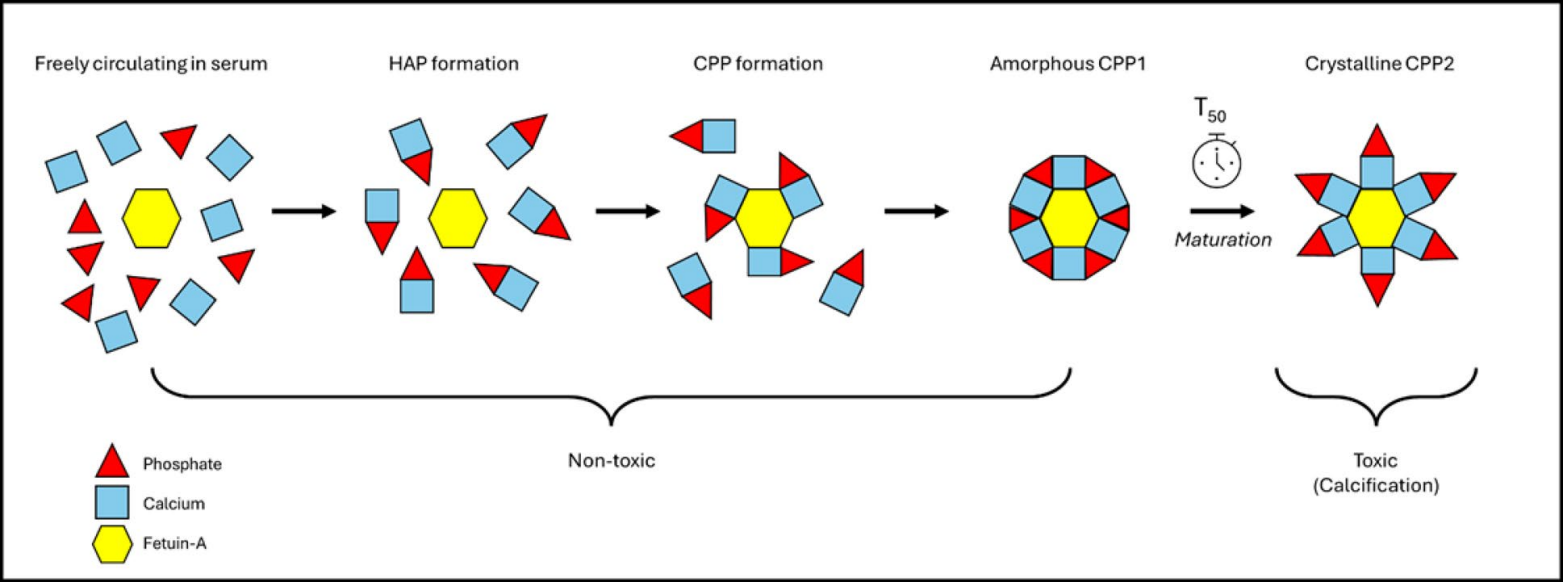
Concept of the T_50_ calciprotein crystallization test. In serum, freely circulating phosphate and calcium ions spontaneously aggregate and form hydroxyapatite crystals (HAP). These crystals bind to calcification-inhibiting molecules, mainly fetuin-A, to form primary amorphous calciprotein particles (CPP1). CPP1 molecules undergo maturation to secondary crystalline calciprotein particles (CPP2), which are responsible for arterial calcification. The T_50_ captures the one-half maximum transition time for CPP1 to completely transform to CPP2.

In 2012, Pasch et al. introduced the T_50_ calciprotein crystallization test as a potential biomarker for CVD risk, including arterial calcification [6]. It is based on the concept that serum phosphate and calcium spontaneously aggregate, and subsequently bind to fetuin-A to form primary calciprotein particles (CPP1). The amorphous CPP1 undergo maturation to secondary crystalline CPP2, which are involved in arterial calcification. The T_50_ is measured by nephelometry and represents the one-half maximum transition time for CPP1 to completely transform to CPP2. The T_50_, expressed in minutes, is therefore a measure of the endogenous ability to prevent precipitation of calcium phosphate in serum, with a higher (slower) T_50_ indicating more calcification protection (Fig. 1).

Observational studies have investigated the potential of T_50_ as biomarker of arterial calcification and CVD risk. High T_50_ was inversely associated with aortic calcification Agatston score and atherosclerotic CVD score in patients with primary aldosteronism [7], and with coronary artery calcium (CAC) score in chronic kidney disease (CKD) patients [8]. A Dutch study observed that every 60-min decrease in T_50_ was associated with increased hazard of CVD-mortality and all-cause mortality in individuals with T2DM [9]. Finally, low T_50_ was associated with higher risk of major adverse cardiovascular events (MACE) and cardiovascular mortality in individuals with PAD [10]. However, the association of T_50_ with other CVD risk markers, especially with regard to common CVD-manifestations in T2DM such as HFpEF and PAD, has not been investigated to date.

In this study, we aimed to investigate the association between T_50_ and (1) HFpEF (2) ankle-brachial index (ABI) as measure of PAD (3) carotid-femoral pulse wave velocity (cfPWV) as measure of central arterial stiffness and (4) arterial calcification in individuals aged 50–75 years with T2DM in a primary care setting. This research will shed light on the applicability of T_50_ as potential biomarker of various surrogate CVD risk markers.

## Methods

### Study design

Cross-sectional data was used of 844 individuals from the Diabetes Care System (DCS) cohort. The DCS cohort consists of over 15,000 individuals with T2DM from the West-Friesland region in the Netherlands. Participants were referred to the DCS study centre by their general practitioner for annual monitoring of glycaemic control, diabetes-related risk factors and complications in the period 1998–2019. They visited the study centre triennially in the period 2020–2022. Details of the DCS and T2DM confirmation are described elsewhere [11]. DCS participants who provided written informed consent to be contacted for future research were invited to participate in the Early-HFpEF study (2019–2023) with the aim to collect data on arterial calcification and left ventricular diastolic function [12]. The Early-HFpEF study was conducted in agreement with the principles of the Declaration of Helsinki and the Medical Research Involving Human Subjects Act (WMO). Medical ethical approval was obtained (METC VUmc, 2018.064—EARLY-HFPEF).

### Study population

Eligible individuals were 50–75 years old and diagnosed with T2DM at least one year before recruitment and were able to provide written informed consent. Participants with severe diabetes complications (e.g. proliferative retinopathy, disabling polyneuropathy or stages IV-V diabetic nephropathy), cardiac comorbidity (e.g. prior diagnosis of heart failure, hypertrophic obstructive cardiomyopathy or congenital heart disease) or severe non-cardiac comorbidity (e.g. severe hepatic insufficiency, severe renal dysfunction or malignancy) were excluded.

### *T*_*50*_* calciprotein crystallization test*

T_50_ was measured using the T_50_ calciprotein crystallization test on non-fasting serum samples, which were frozen and stored at  − 80^ °^C in the Amsterdam UMC biobank. After study completion, the frozen serum samples were shipped to Calciscon AG (Biel, Switzerland) for determination of T_50_ via nephelometry, as described by Pasch [6]. Frozen samples are suitable for this assay in a research setting and the coefficient of variation was 6% [6].

### Computed tomography (CT)

An unenhanced Somatom Definition AS 64-slice CT scan (Siemens Healthcare, Erlangen, Germany) was performed in all participants of the lower-extremities and coronary arteries at 120 kilovolt peak (spatial resolution 0.33 mm, reconstructed at slice thickness 3 mm, increment 1.5 mm). The lower extremities included the femoral and crural arterial beds. The femoral artery was defined as superficial femoral artery and popliteal artery and crural artery was defined as lower leg arteries from the trifurcation until the ankle joint. ECG-gating was performed to correct for movement during scanning of the coronary arteries. The calcification score was measured in the femoral, crural and coronary arteries using a locally developed software tool (iX Viewer, Utrecht University Medical Centre) and the Agatston method [13]. The femoral and crural calcification scores bed were summed to a total peripheral calcification score. Attributable to the extreme right-skewedness of calcification scores, these variables were categorized as 0 (reference category) and tertiles above 0. Details regarding the calcification measurements and scoring are described elsewhere [12].

### Echocardiography and definition of HFpEF

Echocardiography was performed using transthoracic echocardiography with a Philips Affiniti 70 Ultrasound System and a Pure Wave S5-1 transthoracic transducer (Philips, Eindhoven, the Netherlands). HFpEF was defined using the European Society of Cardiology (ESC) 2021 guidelines [14]. This diagnosis includes (1) subjective signs or symptoms of heart failure (exertional dyspnoea, orthopnoea and/or peripheral oedema), (2) left ventricular ejection fraction (LVEF) ≥ 50% and (3) objective evidence of cardiac structural and/or functional abnormalities consistent with the presence of LVDD and/or increased left ventricular filling pressure, including increased NTproBNP values. Moreover, we also used the Dutch Primary Care (NHG) 2024 guidelines for definition of HFpEF as sensitivity analysis by isolation of the NTproBNP cut-off value from the third criterium and using it as a separate fourth criterium. For details regarding echocardiography and HFpEF diagnosis, see Supplementary Files part A and B.

### Vascular physiology

The ABI was calculated by measuring the systolic blood pressures at the brachialis, dorsalis pedis and posterior tibialis in duplicate using a Vasoguard Doppler probe (8 MHz). The ABI was calculated separately for the dorsalis pedis and posterior tibialis by dividing the average ankle systolic blood pressure (SBP) by the highest average arm SBP at the same side of the participant. Individuals with ABI ≤ 0.9 were considered to have occlusive PAD. The cuff was not inflated beyond 220 mmHg in order to prevent discomfort. Individuals with SBP ≥ 220 mmHg in the ankles were considered to have incompressible arteries and thus ABI ≥ 1.4. The ABI was categorized as ≤ 0.9 (PAD), 0.9–1.4 (normal) and ≥ 1.4 (high ABI i.e. incompressible arteries). In a sensitivity analysis, ABI ≥ 1.3 was used as cut-off value. Subsequently, the SBP and diastolic blood pressure (DBP) were measured in supine position in triplicate using an automated oscillomat (SphygmoCor) with 30-s intervals. Finally, the cfPWV, a measure of central arterial stiffness, was measured in duplicate using the same oscillomat. A third measurement was conducted when two consecutive cfPWV measurements differed more than 1.0 m/s. Mean values were computed for analysis.

### Biochemical measurements

Anticoagulated EDTA blood samples were drawn for immunoassay-based analysis of NT-proBNP (pg/mL) performed by U-diagnostics (Baarn, the Netherlands). Data on other biochemical markers were collected via linkage to the DCS routine care database when data were available within one year of the study visit, otherwise through analysis of serum collected during the study visit for biobank purposes. These biochemical markers included total cholesterol, HDL-cholesterol, LDL-cholesterol, triglycerides (all mmol/L), HbA1c (mmol/mol) and creatinine levels (µmol/L). Data on these markers via both routes were obtained by analysis within the same lab (DiagnostIQ, Hoorn, the Netherlands) using the Capillarys technique for HbA1c and the Cobas 8000 technique for all other markers.

### Covariates

Data on demographics, medication use and smoking were obtained by linkage to DCS routine care data from the visit closest to the study date. Demographic variables included age (years), sex (male/female) and duration of diabetes (years). Lifestyle variables included BMI (via self-reported height and weight; kg/m^2^), hypertension (yes/no), hypercholesterolemia (yes/no), smoking status (self-reported; ever/never) and alcohol intake (self-reported; non-drinker/drinker). The SBP (≥ 140 mmHg), DBP (≥ 90 mmHg) and prescription of antihypertensive medication (yes/no; drugs with ATC codes C02, C03, C07, C08 and C09) were used to define hypertension (yes/no) if at least one of these three criteria were fulfilled. Hypercholesterolemia was dichotomously defined as total cholesterol ≥ 5.0 mmol/L, LDL-cholesterol ≥ 3.2 mmol/L and/or using lipid-lowering medication (yes/no; ATC code C10). Finally, the glomerular filtration rate (eGFR; ml/min/1.73 m^2^) was estimated with the CKD-EPI formula [15].

Medical variables included history of CVD (yes/no; coronary artery disease and/or cerebrovascular disease), CVD-related drug use (yes/no; ATC code C), insulin use (yes/no; ATC code A10A) and non-insulin glucose-lowering drugs (yes/no; ATC code A10B). History of CVD was obtained via questionnaires. All participants in this cohort used glucose-lowering medication so the covariate regarding insulin use distinguishes participants using insulin from participants using oral glucose-lowering medication.

### Statistical analysis

Baseline characteristics were described for the full cohort and per category of T_50_ (≤ 330, 330–390, ≥ 390 min). Continuous variables were expressed as mean (standard deviation) or median (interquartile range) depending on their distribution. Categorical variables were presented as frequency (percentage).

First, we investigated the association of T_50_ with HFpEF using multivariable-adjusted Poisson regression analysis since the outcome did not comply with the rare outcome assumption of binary logistic regression analysis, and odds ratios (OR) overestimate risk ratios (RR) when the event is common [16]. The effect estimates consisted of RR and 95% confidence intervals (CI). As sensitivity analysis, we used the NHG-2024 definition of HFpEF using logistic regression analysis.

Second, we evaluated the association between T_50_ and ABI using a multivariable-adjusted multinomial regression analysis with normal ABI as reference category. As sensitivity analysis, we used ABI ≥ 1.3 as cut-off value for high ABI i.e. incompressible arteries.

Third, we evaluated whether T_50_ was associated with cfPWV using multivariable-adjusted linear regression analysis. The effect estimates consisted of regression coefficients (β) and 95%-CI. Standard assumptions of linear regression were checked.

Finally, we investigated whether T_50_ was associated with arterial calcification using two separate multivariable-adjusted multinomial regression analyses with each different outcomes (1) peripheral calcification score, and (2) coronary calcification score. Zero calcification was regarded as outcome reference category.

All analysis were performed with T_50_ as continuous and as categorical variable. The T_50_ was categorized as ≤ 330 min, 330–390 min and ≥ 390 min (reference category) based on its distribution within the study sample to approximate tertiles. The median values within each category approximate 300, 360 and 420 min, respectively, and so roughly 60-min differences were studied. We checked for linearity of the association by adding a quadratic T_50_ variable to the model. We provided the *p*-for-trend for the categorical analyses.

Confounders were selected based on literature. Model 1 included adjustment for demographics (age, sex, and diabetes duration). Model 2 included adjustment for model 1 + lifestyle variables (BMI, hypertension, hypercholesterolemia, smoking status, alcohol intake, and eGFR). Model 3 included adjustment for model 2 along with medical variables (history of CVD, HbA1c, and insulin use). Attributable to their non-linear relationships with the outcomes, the following variables were categorized: duration of diabetes (< 14; >  = 14 years), BMI (< 30; >  = 30 kg/m^2^), eGFR (< 90; >  = 90 ml/min/1.73 m^2^) and HbA1c (< 52; 52–62; > 62 mmol/mol).

Effect modification by sex, smoking status and history of CVD were checked by adding an interaction term to model 3. If the *p*-for-interaction values were below 0.1, the analysis was stratified accordingly.

We performed two additional sensitivity analyses. First, we repeated the main analyses with T_50_ operationalized in tertiles to evaluate whether data-driven partitioning of T_50_ affected observed associations (Supplementary Files part D), and second, we conducted complete case analysis to evaluate whether data imputation affected observed associations (Supplementary Files part E).

We used multiple imputation (80 datasets, 20 iterations) as the percentage of missing data for multiple variables was above 5%. All exposure variables and covariates were used as predictors. For HbA1c, we additionally used HbA1c level in 2019 as predictor.

All analyses were carried out using R software (Version 4.3, RStudio, Boston, Massachusetts, USA). A probability value of 0.05 was used as cut-off value to assess statistical significance, unless stated otherwise.

## Results

### Baseline characteristics

**Fig. 2:**
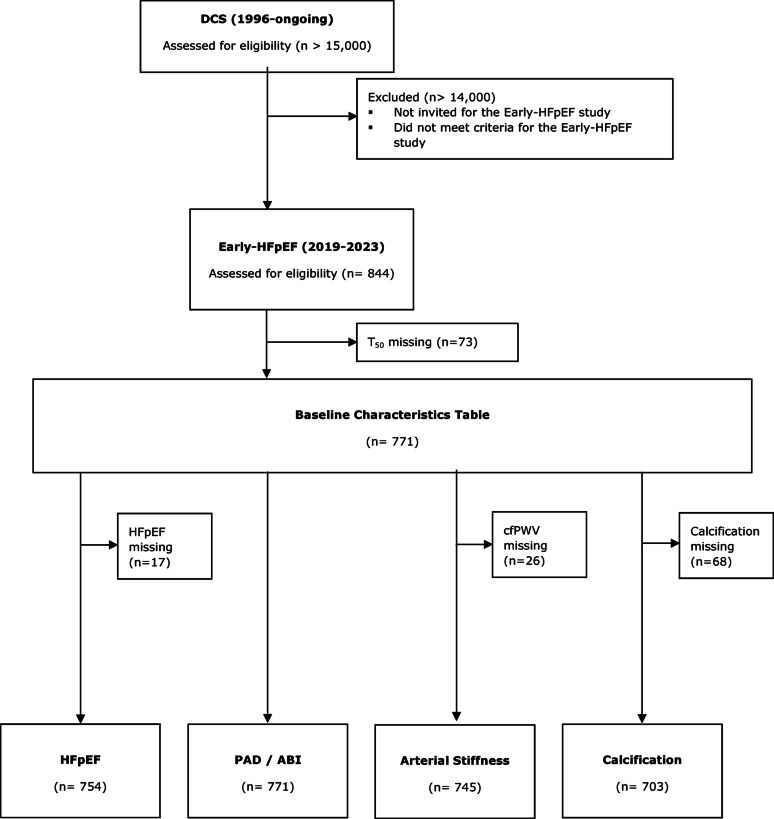
Flow diagram of participants of the diabetes care system (DCS) invited for the Early-HFpEF cohort study. Participants with complete data on T_50_ were included in the baseline characteristics table. Next, four separate datasets were created for the analyses regarding heart failure with preserved ejection fraction (HFpEF), peripheral artery disease (PAD) / ankle-brachial index (ABI), arterial stiffness and arterial calcification.

T_50_ data was available in 771 participants. Four datasets were created for the analyses regarding HFpEF (n = 754), ABI/PAD (n = 771), arterial stiffness (n = 745) and arterial calcification (n = 703) (Fig. 2). Median age was 67 [63–71] years and 64% were male (Table 1). The participants had a median diabetes duration of 15 [11–18] years and 24% reported a history of CVD. T_50_ was 355 ± 55 min. Individuals with lower T_50_ were more often female and alcohol drinkers, had a higher NTproBNP value, had less often a history of cerebrovascular disease, appeared to have more calcification and tended to have a lower ABI (Table 1).

**Table 1:** Baseline characteristics for the full cohort and stratified on T_50_ category (≤ 330, 330–390 and ≥ 390 min).

Baseline characteristics per T_50_ category	*Total cohort* *(n* = *771)*	*T*_*50*_ ≤ *330 min* *(high calcification formation rate)* *(n* = *245)*	*330* < *T*_*50*_ < *390 min* *(medium calcification formation rate)* *(n* = *317)*	*T*_*50*_ ≥ *390 min* *(low calcification formation rate)* *(n* = *209)*
*Demographic variables*				
Age (years)	67 (63–71)	67 (63–70)	68 (63–72)	67 (62–70)
Male sex (yes)	490 (63.6)	148 (60.4)	195 (61.5)	147 (70.3)
Duration of diabetes (years)	15 (11–18)	15 (12–19)	14 (11–18)	15 (11–18)
*Lifestyle variables*				
BMI (kg/m^2^)	29.7 ± 4.9	29.2 ± 5.1	30.0 ± 4.8	29.7 ± 4.7
Hypertension (yes)^§^	655 (92.6)	202 (93.1)	278 (93.0)	175 (91.6)
Hypercholesterolemia (yes)^§^	630 (90.6)	201 (92.6)	265 (90.1)	164 (89.1)
Smoking (ever)^§^	511 (67.4)	156 (64.7)	216 (69.0)	139 (68.1)
Alcohol drinker (yes)^§^	470 (69.7)	158 (74.2)	190 (68.3)	122 (66.7)
*CVD*				
HFpEF based on ESC-2021 (yes)^§^	276 (36.6)	87 (36.0)	114 (36.9)	75 (36.9)
HFpEF based on NHG-2024 (yes)^§^	94 (12.5)	26 (11.0)	47 (15.0)	21 (10.3)
Cerebrovascular disease (yes)^§^	40 (5.9)	6 (2.8)	19 (6.8)	15 (8.2)
Coronary artery disease (yes)^§^	96 (14.2)	29 (13.6)	46 (16.5)	21 (11.5)
*CVD Biomarkers*				
NT-proBNP (pg/ml)^§^	66 (34–137)	68 (36–147)	77 (35–154)	58 (30–114)
LDL-cholesterol (mmol/L)^§^	2.1 (1.7–2.8)	2.2 (1.7–3.0)	2.1 (1.7–2.8)	2.1 (1.7–2.8)
HDL-cholesterol (mmol/L)^§^	1.1 (1.0–1.4)	1.2 (1.0–1.4)	1.2 (1.0–1.4)	1.1 (0.9–1.4)
Total cholesterol (mmol/L)^§^	4.1 (3.6–4.9)	4.2 (3.6–5.0)	4.1 (3.6–4.9)	4.1 (3.6–4.8)
Triglycerides (mmol/L)^§^	1.9 (1.4–2.7)	1.8 (1.2–2.7)	1.9 (1.4–2.7)	1.9 (1.4–2.6)
eGFR (ml/min/1.73m^2^)^§^	88 (73–96)	90 (73–98)	86 (70–96)	89 (75–96)
HbA1c (mmol/mol)^§^	56 (49–66)	59 (50–67)	54 (49–62)	55 (49–70)
*Medication use*				
Insulin (yes)^§^	232 (36.5)	88 (41.7)	85 (32.3)	59 (36.6)
Non-insulin glucose-lowering (yes)^§^	597 (94.0)	196 (92.9)	249 (94.7)	152 (94.4)
Anti-hypertensive (yes)^§^	515 (80.7)	163 (84.5)	216 (78.8)	136 (79.5)
Lipid-lowering (yes)^§^	520 (81.5)	160 (82.9)	220 (80.3)	140 (81.9)
*Vascular phenotyping*				
Peripheral calcification (Agatston)^§^	791 (64–3820)	1259 (179–4091)	732 (15–3727)	671 (57–3381)
Coronary calcification (Agatston)^§^	315 (40–1246)	368 (52–1281)	322 (33–1422)	240 (27–1115)
Low ABI (≤ 0.9; yes)*†*	45 (5.8)	19 (7.8)	19 (6.0)	7 (3.3)
High ABI (≥ 1.4; yes)	24 (3.1)	12 (4.9)	5 (1.6)	7 (3.3)
High ABI (≥ 1.3; yes)	60 (7.8)	18 (7.3)	26 (8.2)	16 (7.7)
cfPWV (m/s)^§^	12.9 ± 2.5	12.8 ± 2.3	12.9 ± 2.7	13.0 ± 2.2

ABI = Ankle-Brachial Index, BMI = Body Mass Index, cfPWV = Carotid-Femoral Pulse Wave Velocity, CVD = Cardiovascular Disease, eGFR = Estimated Glomerular Filtration Rate, ESC = European Society of Cardiology, HbA1c = Glycated haemoglobin, HDL = High Density Lipoprotein, HFpEF = Heart Failure with Preserved Ejection Fraction, LDL = Low Density Lipoprotein, NHG = Dutch Primary Care, NTproBNP = N-terminal pro-B-type natriuretic peptide, T_50_ = Calciprotein Crystallization Time.

^§^ Missings: hypertension (n = 64), hypercholesterolemia (n = 76), smoking (n = 13), alcohol drinking (n = 97), HFpEF based on ESC-2021 (n = 17), HFpEF based on NHG-2024 (n = 18), cerebrovascular disease (n = 96), coronary artery disease (n = 96), NT-proBNP (n = 17), LDL-cholesterol (n = 5), HDL-cholesterol (n = 4), total cholesterol (n = 4), triglycerides (n = 4), eGFR (n = 5), HbA1c (n = 510), insulin (n = 136), non-insulin glucose-lowering (n = 136), antihypertensive (n = 133), lipid-lowering (n = 133), peripheral calcification (n = 65), coronary calcification (n = 37) and cfPWV (n = 26).

^†^ Equal to peripheral artery disease (PAD).

### HFpEF

The prevalence of HFpEF based on the ESC-2021 guideline was 36.6% (Table 1). Adjusted according to model 3, T_50_ was not associated with HFpEF (per 60 min decrease: RR = 1.02 [0.90–1.17], *p* = 0.736) (Table 2). Using the NHG-2024 definition, the prevalence of HFpEF was 12.5%. Also using this definition, no association was observed between T_50_ and HFpEF (per 60 min decrease: OR = 1.15 [0.89–1.49], *p* = 0.274) (Table 2). No effect modification by sex, smoking status or history of CVD was observed (*p*-for-interaction > 0.251).

**Table 2:** Associations between T_50_ with HFpEF, the ankle-brachial index and central arterial stiffness.

		HFpEF		HFpEF		Ankle-brachial index				Arterial stiffness	
CVD OUTCOMES		*(based on ESC-2021)*		*(based on NHG-2024)*		≤ *0.9 (PAD) *^*(b)*^		≥ *1.4 *^*(b)*^		*(cfPWV; continuous)*	
		*RR*	*p*	*OR*	*p*	*OR*	*p*	*OR*	*p*	*β*	*p*
Per 60 min decrease	Model 1 ^(c)^	1.00 [0.87–1.13]	*0.953*	1.12 [0.88–1.43]	*0.362*	1.42 [1.02–1.98]	*0.037*	1.09 [0.69–1.71]	*0.707*	-0.07 [-0.25–0.11]	*0.437*
	Model 2 ^(d)^	1.03 [0.90–1.17]	*0.682*	1.17 [0.90–1.51]	*0.233*	1.41 [1.00–1.99]	*0.047*	1.12 [0.70–1.78]	*0.643*	-0.08 [-0.26–0.11]	*0.413*
	Model 3 ^(e)^	1.02 [0.90–1.17]	*0.736*	1.15 [0.89–1.49]	*0.274*	1.46 [0.97–2.21]	*0.072*	1.11 [0.69–1.78]	*0.665*	-0.08 [-0.26–0.10]	*0.398*
≤ 330 min^(a)^	Model 1 ^(c)^	0.92 [0.67–1.25]	*0.588*	0.99 [0.53–1.83]	*0.964*	2.56 [1.05–6.27]	*0.039*	1.73 [0.66–4.53]	*0.263*	-0.06 [-0.49–0.37]	*0.780*
	Model 2 ^(d)^	1.01 [0.74–1.39]	*0.932*	1.13 [0.59–2.13]	*0.714*	2.62 [1.05–6.53]	*0.039*	1.76 [0.66–4.71]	*0.260*	-0.07 [-0.50–0.36]	*0.749*
	Model 3 ^(e)^	1.00 [0.73–1.37]	*0.996*	1.10 [0.58–2.08]	*0.777*	3.04 [1.03–8.94]	*0.044*	1.77 [0.66–4.77]	*0.259*	-0.08 [-0.51–0.35]	*0.718*
330–390 min^(a)^	Model 1 ^(c)^	0.94 [0.70–1.25]	*0.657*	1.37 [0.78–2.40]	*0.270*	1.82 [0.75–4.44]	*0.188*	0.48 [0.15–1.56]	*0.224*	-0.04 [-0.45–0.36]	*0.841*
	Model 2 ^(d)^	0.96 [0.71–1.28]	*0.765*	1.42 [0.80–2.52]	*0.229*	1.78 [0.72–4.38]	*0.212*	0.48 [0.15–1.58]	*0.227*	-0.05 [-0.45–0.36]	*0.818*
	Model 3 ^(e)^	0.96 [0.71–1.28]	*0.766*	1.38 [0.78–2.47]	*0.270*	1.59 [0.57–4.47]	*0.379*	0.46 [0.14–1.52]	*0.203*	-0.02 [-0.43–0.38]	*0.904*

T_50_ was operationalized per 60-min decrease and as categorical variable. HFpEF was based on ESC-2021 and NHG-2024 guidelines. The ABI was categorized as ≤ 0.9 (PAD), 0.9–1.4 (normal, reference category) and ≥ 1.4 (high). Central arterial stiffness was continuously measured per 1 m/s increment. Values within square brackets are the 95%-C.I.

cfPWV = Carotid-femoral Pulse Wave Velocity, C.I. = Confidence Interval, CVD = Cardiovascular Disease, ESC = European Society of Cardiology, HFpEF = Heart Failure with preserved Ejection Fraction, NHG = Dutch Primary Care, OR = Odds Ratio, PAD = Peripheral Artery Disease, RR = Risk Ratio.

(a) The reference is T_50_ ≥ 390 min.

(b) The reference category is 0.9 < ABI < 1.4.

(c) Adjusted for age (years), sex (male/female) and duration of diabetes (< 14 years / ≥ 14 years).

(d) Adjusted for model 1 + BMI (< 30 kg/m^2^ / ≥ 30 kg/m^2^), hypertension (yes/no), hypercholesterolemia (yes/no), smoking status (ever/never), alcohol intake (non-drinker/drinker) and eGFR (< 90 mL/min/1.73 m^2^ / ≥ 90 mL/min/1.73 m^2^).

(e) Adjusted for model 2 + insulin drug use (yes/no), history of CVD (yes/no) and HbA1c (< 52 mmol/mol / 52–62 mmol/mol / > 62 mmol/mol).

*p* for linearity values in model 3: 0.848 (HFpEF).

< 0.001, 0.283, 0.005, 0.228, 0.063 (ABI).

0.114 (arterial stiffness).

*p* for trend values in model 3: 0.037 (ABI ≤ 0.9), 0.190 (ABI ≥ 1.4).

### ABI / PAD

PAD (low ABI) was observed in 5.8% and high ABI in 3.1% of the cohort (Table 1). Non-linearity between T_50_ and ABI categories was observed (lowest *p* for quadratic T_50_ < 0.001), and therefore the association was evaluated with T_50_ as categorical variable. T_50_ ≤ 330 min versus T_50_ ≥ 390 min was associated with higher odds of PAD (OR = 3.04 [1.03–8.94], *p* = 0.044), but not with ABI ≥ 1.4 (OR = 1.77 [0.66–4.77], *p* = 0.259) (Table 2; model 3). Moreover, 330 < T_50_ < 390 min was not associated with neither PAD nor ABI ≥ 1.4 (OR = 1.59 [0.57–4.47], *p* = 0.379; OR = 0.46 [0.14–1.52], *p* = 0.203, respectively). The *p*-for-trend values were 0.037 for the outcome ABI ≤ 0.9 and 0.190 for the ABI ≥ 1.4 outcome. Similar observations were observed when using ABI ≥ 1.3 as cut-off value for high ABI (i.e. incompressible arteries) (Supplementary Table 1). Sex, smoking status and history of CVD were not effect modifiers (*p*-for-interaction > 0.481).

### Arterial stiffness

Mean cfPWV was 12.9 ± 2.5 m/s in the total cohort (Table 1). According to model 3, T_50_ was not associated with cfPWV (per 60 min decrease: β =  − 0.08 [ − 0.26–0.10], *p* = 0.398) (Table 2). No effect modification was observed for sex, smoking status and history of CVD (*p*-for-interaction > 0.596).

### Arterial calcification

Median calcification scores were 315 [40–1246] and 791 [64–3820] Agatston units in the coronary and peripheral arteries, respectively (Table 1). Every 60-min decrease in T_50_ was significantly associated with high (OR = 1.63 [1.15–2.30], *p* = 0.006), medium (OR = 1.52 [1.09–2.10], *p* = 0.013) and low (OR = 1.37 [1.00–1.87], *p* = 0.053) amounts of CAC compared to no CAC (Table 3; model 3). In contrast, every 60-min decrease in T_50_ was not associated with high (OR = 1.28 [0.96–1.69], *p* = 0.088), medium (OR = 1.20 [0.92–1.57], *p* = 0.172) and low (OR = 1.07 [0.83–1.37], *p* = 0.612) peripheral calcification compared to no peripheral calcification (Table 3; model 3). In both arterial beds, we observed that the OR increased in magnitude along increasing calcification categories. Moreover, the OR for peripheral arterial calcification, albeit lower in magnitude, had a similar direction as the OR for CAC. Finally, no effect modification was observed for sex, smoking status and history of CVD regarding any of the calcification analyses (lowest *p*-for-interaction = 0.040 (sex on peripheral calcification), all others *p* > 0.222; so no stratification on sex was performed).

**Table 3:** Associations between T_50_ with peripheral arterial calcification score and coronary arterial calcification score.

CALCIFICATION SCORE			*Tertile 1 (low) * ^*(b)*^		*Tertile 2 (medium) * ^*(b)*^		*Tertile 3 (high) * ^*(b)*^	
			*OR*	*p*	*OR*	*p*	*OR*	*p*
Peripheral calcification score	Per 60 min decrease	Model 1 ^(c)^	1.11 [0.87–1.42]	*0.407*	1.23 [0.95–1.59]	*0.109*	1.33 [1.02–1.74]	*0.035*
		Model 2 ^(d)^	1.06 [0.83–1.37]	*0.622*	1.22 [0.94–1.58]	*0.144*	1.32 [1.00–1.73]	*0.050*
		Model 3 ^(e)^	1.07 [0.83–1.37]	*0.612*	1.20 [0.92–1.57]	*0.172*	1.28 [0.96–1.69]	*0.088*
	≤ 330 min ^(a)^	Model 1 ^(c)^	1.23 [0.67–2.26]	*0.495*	1.93 [1.03–3.63]	*0.041*	1.83 [0.95–3.53]	*0.073*
		Model 2 ^(d)^	1.14 [0.62–2.13]	*0.669*	1.98 [1.04–3.76]	*0.038*	1.86 [0.95–3.64]	*0.072*
		Model 3 ^(e)^	1.14 [0.61–2.12]	*0.681*	1.93 [1.01–3.70]	*0.048*	1.76 [0.89–3.51]	*0.106*
	330–390 min ^(a)^	Model 1 ^(c)^	0.74 [0.43–1.29]	*0.292*	0.85 [0.47–1.52]	*0.579*	0.92 [0.50–1.69]	*0.797*
		Model 2 ^(d)^	0.76 [0.43–1.32]	*0.325*	0.85 [0.47–1.53]	*0.579*	0.92 [0.50–1.70]	*0.788*
		Model 3 ^(e)^	0.76 [0.43–1.33]	*0.337*	0.81 [0.45–1.47]	*0.487*	0.84 [0.45–1.57]	*0.587*
Coronary calcification score	Per 60 min decrease	Model 1 ^(c)^	1.36 [1.00–1.85]	*0.048*	1.50 [1.09–2.06]	*0.012*	1.69 [1.22–2.33]	*0.002*
		Model 2 ^(d)^	1.35 [0.99–1.84]	*0.062*	1.50 [1.08–2.07]	*0.014*	1.67 [1.20–2.33]	*0.003*
		Model 3 ^(e)^	1.37 [1.00–1.87]	*0.053*	1.52 [1.09–2.10]	*0.013*	1.63 [1.15–2.30]	*0.006*
	≤ 330 min ^(a)^	Model 1 ^(c)^	1.86 [0.91–3.78]	*0.087*	2.02 [0.98–4.17]	*0.057*	2.76 [1.30–5.83]	*0.008*
		Model 2 ^(d)^	1.79 [0.87–3.70]	*0.113*	2.05 [0.98–4.30]	*0.058*	2.72 [1.26–5.85]	*0.011*
		Model 3 ^(e)^	1.84 [0.89–3.81]	*0.102*	2.08 [0.98–4.40]	*0.055*	2.64 [1.19–5.86]	*0.017*
	330–390 min ^(a)^	Model 1 ^(c)^	2.10 [1.07–4.10]	*0.031*	1.72 [0.86–3.43]	*0.126*	2.22 [1.08–4.55]	*0.029*
		Model 2 ^(d)^	2.18 [1.11–4.29]	*0.024*	1.75 [0.87–3.53]	*0.115*	2.24 [1.08–4.62]	*0.029*
		Model 3 ^(e)^	2.20 [1.12–4.35]	*0.023*	1.72 [0.85–3.49]	*0.130*	2.00 [0.94–4.24]	*0.072*

T_50_ was operationalized per 60-min decrease and as categorical variable. Peripheral arterial calcification score and coronary arterial calcification score were both operationalized into zero + tertiles > 0. Values within square brackets are the 95%-C.I.

C.I. = Confidence Interval, OR = Odds Ratio.

(a) The reference is T_50_ ≥ 390 min.

(b) The reference is no calcification (score = 0).

(c) Adjusted for age (years), sex (male/female) and duration of diabetes (< 14 years / ≥ 14 years).

(d) Adjusted for model 1 + BMI (< 30 kg/m^2^ / ≥ 30 kg/m^2^), hypertension (yes/no), hypercholesterolemia (yes/no), smoking status (ever/never), alcohol intake (non-drinker/drinker) and eGFR (< 90 mL/min/1.73 m^2^ / ≥ 90 mL/min/1.73 m^2^).

(e) Adjusted for model 2 + insulin drug use (yes/no), history of CVD (yes/no) and HbA1c (< 52 mmol/mol / 52–62 mmol/mol / > 62 mmol/mol).

*p* for linearity values in model 3: 0.006, 0.534, < 0.001, 0.425, < 0.001, 0.417 (peripheral calcification score) 0.441, 0.975, 0.001, 0.228, < 0.001, 0.463 (coronary calcification score).

Range Scores Peripheral Coronary.

Zero [0] [0].

Tertile 1 [1–694] [1–163].

Tertile 2 [695–3,538] [164–936].

Tertile 3 [3539–66,318] [937–23,436].

### Sensitivity analyses

We observed no remarkable differences in observed associations between the original study results and results obtained from T_50_ tertile operationalization (Supplementary Tables S2 and S3) and obtained from complete case analysis (Supplementary Tables S4 and S5).

## Discussion

This study demonstrated that in a primary care setting of individuals aged 50–75 years with T2DM and a relatively high T_50_, a low T_50_ was associated with an increased risk of PAD and higher CAC, but not with HFpEF, ABI ≥ 1.4, central arterial stiffness and peripheral calcification.

The observed T_50_ was higher compared to two Dutch T2DM studies in a primary care setting (mean T_50_ 261 ± 66 min), [17] and in in a secondary care setting (mean T_50_ 323 ± 63 min) [9]. In contrast, a Dutch T2DM trial with similar age- and sex distribution reported a similar T_50_-distribution of 350 [320–395] minutes [18]. These varying distributions may be attributable to varying renal function or glycaemic control within these cohorts, which are relatively well managed in the DCS cohort.

First, we observed that low T_50_ was associated with CAC, but not with peripheral arterial calcification. The first finding is in agreement with previous literature on T_50_ and CAC in CKD patients (median T_50_ 321 [270–366] minutes) [8]. To the best of our knowledge, there is no literature on T_50_ and peripheral calcification. Similar observations were observed within the aorta [7]. Based on a previous study, we assumed that calcification is independent of the arterial bed involved [19]. Indeed, it should be noted that the *p*-value for the highest calcification tertile was just short of statistical significance (*p* = 0.088) in model 3 and it was < 0.05 in models 1 and 2. Together with the increasing ORs along increasing calcification tertiles and direction of the odds ratios, we speculate that there is a weak and inverse association between T_50_ and peripheral arterial calcification. A possible explanation for the null result is reduced accuracy of the peripheral arterial calcification measurement due to larger arterial territory in combination with image distortion due to scatter. Moreover, calcification score is a result of build-up, whereas the T_50_ captures an instantaneous CPP2 formation rate. It is conceivable that the T_50_ varies over time and so T_50_ at a certain moment does not reflect the calcification formation over the years preceding the T_50_ measurement moment. We hypothesize that the T_50_ decreases with age, yet evidence is lacking in this population. However, a decline in T_50_ has been demonstrated in haemodialysis patients during a two-year follow-up [20]. Moreover, it might be possible that the T_50_ represents a more complex system than just CPP1 maturation to CPP2. As such, we speculate that the null-association for peripheral calcification is a false negative finding due to validity issues. Future research should focus on longitudinal associations between T_50_ and arterial calcification.

Next, low T_50_ was associated with low ABI, but not high ABI. The results regarding high ABI are corroborated by studies in haemodialysis patients [21]. In contrast, a Dutch study investigated the association between low T_50_ and PAD in the general population (mean T_50_ 329 ± 58 min), showing no significant association [22]. The discrepancy may be attributable to the method for defining PAD, which was not based on ABI ≤ 0.9, but on the history of vascular interventions. A possible explanation for our finding is that ABI ≤ 0.9 and ABI ≥ 1.4 may represent two different entities, with a low ABI resulting from obstruction due to lower-extremity arterial calcification and a high ABI possibly due to stiff leg arteries as a result of fibrosis or degradation of elastin fibres. Previous research demonstrated that an ABI ≥ 1.4, suggestive of stiff peripheral arteries, is not a proxy for arterial calcification in the tunica media [23], suggesting that other factors may cause ABI ≥ 1.4. T_50_ was indeed not associated with PWV in our study, lending support to this hypothesis. The odds ratios for the association between T_50_ and PAD may be biased by the low power due to the low prevalence of PAD (5.8%).

The study results regarding HFpEF are in agreement with previous research on T_50_ and heart failure in CKD patients [24], in patients with T2DM [9], and in the general population [25]. Notably, all referred studies used heart failure as outcome instead of HFpEF. Nevertheless, the data collectively argue against an association with HFpEF. As such, we did not expect an association between T_50_ and HFpEF, and this hypothesis was therefore confirmed.

Our results regarding absence of association between T_50_ and arterial stiffness are in agreement with an American study of haemodialysis patients (mean T_50_ 317 ± 22 min), [21]. Moreover, that study reported that T_50_ was not associated with CAC, contradicting our findings. Moreover, low T_50_ was associated with higher baseline cfPWV and cfPWV progression in predialysis patients (baseline T_50_ 329 ± 95 min), [26] which opposes our findings. An explanation for our null finding is that arterial stiffness was not caused by arterial calcification, but by fibrosis or degradation of elastin fibres, as suggested previously [23]. To conclude, the existing literature is conflicting and warrants further investigation of the underlying pathophysiology of arterial stiffness in T2DM.

Strengths of this study were the use of a clinically relevant population at high risk of CVD due to T2DM, the use of multiple forms of CVD and the elaborate procedures to obtain data via echocardiography, pulse wave velocity and computed tomography. Limitations of this study were the cross-sectional study design, which precludes causal inference, and the missing data on HbA1c and arterial calcification due to the COVID-19 pandemic. In addition, we did not collect data on treatments that alter T_50_ (such as vitamin K, magnesium and phosphate binders), calcium-phosphate parameters, diet, physical activity, and inflammation, possibly leading to confounding bias. Next, we grouped glucose-lowering medication as insulin versus non-insulin (oral), which may confound CVD outcomes. Additionally, validity issues may originate from the T_50_ measurements and peripheral arterial calcification quantification, as discussed previously. Moreover, cardiac outcomes were limited to early HFpEF, that were subclinical, due to exclusion of known heart failure cases and cardiomyopathies at baseline. This is supported by the low levels of NTproBNP among HFpEF cases (88 [38–174] pg/mL). Inclusion of heart failure with reduced ejection fraction (HFrEF) cases may be useful as HFrEF has a calcification pathway and should be considered for future research. Similarly, hard outcomes such as coronary revascularization, myocardial infarction and stroke may be included. Finally, the generalizability of the study is limited due to the use of a single regional relatively healthy T2DM cohort, consisting of individuals aged 50–75 years from the West-Friesland region who typically have European ancestry, which hampers the translation to younger individuals, individuals with other ancestries and individuals with severe diabetes or CVD complications.

Nearly half of all individuals with T2DM die of CVD. Arterial calcification is assumed to contribute to this mortality risk. These calcifications are detected in clinical practice using CT, but its applicability is limited because of their high costs, labour intensity and patient burden. The T_50_ may provide as a low-cost and relatively safe independent risk factor for arterial health. The T_50_ reflects the endogenous ability to prevent precipitation of calcium phosphate in serum, with lower T_50_ indicating less calcification protection due to disturbed mineral metabolism, leading to faster CPP2 formation and thus higher CVD risk. Future large scale longitudinal studies are warranted with repeated measurements of T_50_ next to traditional CVD risk factors (age, sex, SBP, LDL-cholesterol, et cetera.) to assess added predictive value of T_50_ with regard to hard CVD outcomes, such as CVD-mortality, PAD, myocardial infarction and stroke or composite outcomes such as MACE. It is conceivable that, once validated, T_50_ measurements may be added to the set of biochemical markers for T2DM routine care check-up visits in primary care, in which a low T_50_ is indicative of higher risk of developing CVD, especially arterial calcification or obstructive PAD. Next, these individuals require more intensive screening for CVD than individuals with higher T_50_. Moreover, interventional strategies can be targeted at increasing T_50_. In a previous trial, we could not increase T_50_ with vitamin K [18].

## Conclusions

To conclude, low T_50_ was associated with ABI ≤ 0.9, indicative of obstructive PAD, and with CAC in individuals with T2DM in a primary care setting. T_50_ was not associated with HFpEF, ABI ≥ 1.4, arterial stiffness and lower-extremity arterial calcification. Further research is needed to evaluate the additive value of T_50_ in CVD risk stratification in clinical care.

## Supplementary Information

Below is the link to the electronic supplementary material.

Supplementary Material 1

## Data Availability

Data can be available upon reasonable request. Data request can be sent to The Hoorn Studies Steering Committee at Hoornstudy@vumc.nl. More information regarding the procedures for contact can be found on www.hoornstudies.com.

## Abbreviations

ABI: Ankle-brachial index
ATC: Anatomical therapeutical classification
BMI: Body mass index
CPP: Calciprotein crystallization time
CAC: Coronary arterial calcification
cfPWV: Carotid-femoral pulse wave velocity
CI: Confidence interval
CKD: Chronic kidney disease
CT: Computed tomography
CVD: Cardiovascular disease
DBP: Diastolic blood pressure
DCS: Diabetes care system
eGFR: Estimated glomerular filtration rate
ESC: European society of cardiology
HbA1c: Glycated haemoglobin
HDL: High-density lipoprotein
HFpEF: Heart failure with preserved ejection fraction
HR: Hazard ratio
LDL: Low-density lipoprotein
LVEF: Left ventricular ejection fraction
MAC: Medial arterial calcification
MACE: Major adverse cardiovascular events
NHG: Dutch primary care
OR: Odds ratio
PAD: Peripheral artery disease
PWV: Pulse wave velocity
RR: Risk ratio
SBP: Systolic blood pressure
T2DM: Type 2 diabetes mellitus
T_50_: Calciprotein crystallization time
WMO: Medical research involving human subjects act
