# Supplementary material for: Calciprotein crystallization time (T50) and its association with surrogate cardiovascular disease risk markers in individuals with type 2 diabetes mellitus: the cross-sectional EARLY-HFpEF study
Source: Cardiovasc Diabetol. 2025 Dec 10;25:10. doi: 10.1186/s12933-025-03016-9 (PMC12801761; doi:10.1186/s12933-025-03016-9)
Supplement: Supplementary file 1 — Supplementary Material 1 [file 12933_2025_3016_MOESM1_ESM.docx]

**Calciprotein crystallization time (T_50_) and its association with cardiovascular outcomes in individuals with type 2 diabetes mellitus: The cross-sectional EARLY-HFpEF Study**

***Supplementary Files***

*A. Echocardiography and Definition of HFpEF*

Mitral inflow early (E) and late (A) were recorded using pulse wave Doppler. Pulsed wave tissue Doppler echocardiography was used to measure peak velocities at the lateral and septal mitral anulus. Peak early diastolic mitral annular velocity (e’), peak systolic velocity (s’) and peak atrial velocity (a’) were calculated as the average peak velocities. The E/e’ ratio was calculated as the ratio of the peak early diastolic mitral inflow (average of the lateral and septal velocities) to the mitral annular early diastolic velocity. The peak velocity of the tricuspid regurgitation (TR) signal was measured in the parasternal right ventricular inflow, parasternal short axis and apical four-chamber views. The Bernoulli equation was used to estimate the right-ventricular-to-right-atrial systolic pressure gradient. The mean right atrial pressure was calculated according to the degree of collapse of the inferior vena cava with inspiration (5 mmHg for a collapse of at least 50% and 15 mmHg for a collapse of less than 50%). The pressure gradient was added to the estimated mean right atrial pressure in order to quantitate the PASP. Left ventricular mass index (LVMI) was calculated from internal dimensions according to the Devereux formula, and indexed to body surface area (BSA). The left atrial (LA) volume was assessed using the biplane area–length method from apical two- and four-chamber views and indexed to BSA resulting in LA volume index (LAVI). Offline analysis was performed by three investigators (AGH, EDC and LH). A set of 20 echograms was scored in twofold and good to excellent inter-observer agreement was observed (ICC ranged between 0.80 for LAVI to 0.99 for e’).

*B. Adapted Kansas City Cardiomyopathy Questionnaire*

Participants were asked to fill out a questionnaire to assess the presence, the severity and the frequency of heart failure symptoms and the resulting physical and social limitation. The questionnaire was adapted from the Kansas City Cardiomyopathy Questionnaire. A person was considered to have symptoms of exertional dyspnoea if he/she reported to experience any degree of limitation in question 1, excluding the final item (“*Hurrying or jogging as if to catch the bus*”). A person was considered to have symptoms of orthopnoea if he/she reported to have been forced to sleep sitting up in a chair or with at least 3 pillows 1-2 days/week or more (question 6). A person was considered to have oedema if he/she reported swelling in the feet, ankles or legs (in the morning) 1-2 days/week or more (question 2) and/or oedema was present during the study visit’s clinical assessment. In case of the participant reporting oedema, researchers AGH and EDC classified the severity as absent/mild/moderate/severe.

1. Please indicate how much you’re limited by shortness of breath or fatigue to do the following activities over the past two weeks.

| **Activity** | **Extremely Limited** | **Quite a bit Limited** | **Moderately Limited** | **Slightly Limited** | **Not at all Limited** | **Limited for other reasons** |
| --- | --- | --- | --- | --- | --- | --- |
| Dressing yourself | □ | □ | □ | □ | □ | □ |
| Showering/Bathing | □ | □ | □ | □ | □ | □ |
| Walking 1 block on level ground | □ | □ | □ | □ | □ | □ |
| Doing yard work, housework or carrying groceries | □ | □ | □ | □ | □ | □ |
| Climbing a flight of stairs without stopping | □ | □ | □ | □ | □ | □ |
| Hurrying or jogging (as if to catch the bus) | □ | □ | □ | □ | □ | □ |

1. Over the past 2 weeks, how many times did you have swelling in your feet, ankles or legs when you woke up in the morning?

| Every morning | 3 or more times a week, but not every day | 1-2 times a week | Less than once a week | Never over the past 2 weeks |
| --- | --- | --- | --- | --- |
| □ | □ | □ | □ | □ |

1. Over the past 2 weeks how bothersome was the swelling in your feet, ankles or legs when you woke up in the morning?

| Extremely bothersome | Quite a bit bothersome | Moderately bothersome | Slightly bothersome | Not at all bothersome | I have no swelling |
| --- | --- | --- | --- | --- | --- |
| □ | □ | □ | □ | □ | □ |

1. Over the past 2 weeks, on average how many times has fatigue limited your ability to do what you want?

| All of the time | Several times per day | At least once a day | 3 or more times per week but not every day | 1-2 times a week | Less than once a week | Never over the past 2 weeks |
| --- | --- | --- | --- | --- | --- | --- |
| □ | □ | □ | □ | □ | □ | □ |

1. Over the past 2 weeks, on average how many times has shortness of breath limited your ability to do what you want?

| All of the time | Several times per day | At least once a day | 3 or more times per week but not every day | 1-2 times a week | Less than once a week | Never over the past 2 weeks |
| --- | --- | --- | --- | --- | --- | --- |
| □ | □ | □ | □ | □ | □ | □ |

1. Over the past 2 weeks, on average how many times have you been forced to sleep sitting up in a chair or with at least 3 pillows?

| Every night | 3 or more times a week but not every day | 1-2 times a week | Less than once a week | Never over the past |
| --- | --- | --- | --- | --- |
| □ | □ | □ | □ | □ |

|  |  | **Ankle-Brachial Index** | | | |
| --- | --- | --- | --- | --- | --- |
| **Sensitivity Analysis** | | *≤0.9 (PAD) ^(b)^* | | *≥1.3 ^(b)^* | |
|  |  | *OR* | *p* | *OR* | *p* |
|  |  |  |  |  |  |
| Per 60 minute increase | Model 1 ^(c)^ | 1.41 [1.02-1.97] | *0.040* | 0.96 [0.72-1.29] | *0.802* |
|  | Model 2 ^(d)^ | 1.41 [1.00-1.98] | *0.050* | 0.97 [0.72-1.32] | *0.860* |
|  | Model 3 ^(e)^ | 1.45 [0.96-2.20] | *0.077* | 0.97 [0.72-1.32] | *0.870* |
|  |  |  |  |  |  |
| ≤330 minutes  ^(a)^ | Model 1 ^(c)^ | 2.54 [1.04-6.20] | *0.041* | 1.17 [0.57-2.38] | *0.674* |
|  | Model 2 ^(d)^ | 2.60 [1.04-6.50] | *0.041* | 1.18 [0.57-2.45] | *0.649* |
|  | Model 3 ^(e)^ | 3.00 [1.02-8.83] | *0.046* | 1.20 [0.58-2.48] | *0.630* |
|  |  |  |  |  |  |
| 330-390 minutes  ^(a)^ | Model 1 ^(c)^ | 1.89 [0.77-4.62] | *0.163* | 1.22 [0.63-2.37] | *0.556* |
|  | Model 2 ^(d)^ | 1.84 [0.74-4.53] | *0.187* | 1.24 [0.63-2.44] | *0.527* |
|  | Model 3 ^(e)^ | 1.67 [0.59-4.71] | *0.330* | 1.22 [0.62-2.40] | *0.563* |
|  |  |  |  |  |  |

*C. Sensitivity Analysis using ABI≥1.3 to define incompressible arteries*

**Table S1:** *Associations between T_50_ with the ankle-brachial index*

*T_50_ was operationalized per 60-minute decrease and as categorical variable. The ABI was categorized as ≤0.9 (PAD), 0.9-1.3 (normal, reference category) and ≥1.3 (high). Values within square brackets are the 95%-C.I..*

*C.I. = Confidence Interval, OR = Odds Ratio, PAD = Peripheral Artery Disease,*

1. *The reference is T_50_≥390 minutes.*
2. *The reference category is 0.9<ABI<1.3.*

*(c) Adjusted for age (years), sex (male/female) and duration of diabetes (<14 years / ≥14 years).*

*(d) Adjusted for model 1 + BMI (<30 kg/m^2^ / ≥30 kg/m^2^), hypertension (yes/no), hypercholesterolemia (yes/no), smoking status (ever/never), alcohol intake (non-drinker/drinker) and eGFR (<90 mL/min/1.73m^2^ / ≥90 mL/min/1.73m^2^).*

*(e) Adjusted for model 2 + insulin drug use (yes/no), history of CVD (yes/no) and HbA1c (<52 mmol/mol / 52-62 mmol/mol / >62 mmol/mol).*

*D. Sensitivity Analysis where T_50_ is operationalized in tertiles*

Tertile 1 (low) n = 262 min 129 median 302 mean 295 max 332

Tertile 2 (medium) n = 254 min 333 median 357 mean 357 max 380

Tertile 3 (high) n = 255 min 381 median 409 mean 415 max 556

|  |  | **HFpEF** | | **HFpEF** | | **Ankle-Brachial Index** | | | | **Arterial Stiffness** | |
| --- | --- | --- | --- | --- | --- | --- | --- | --- | --- | --- | --- |
| **CVD OUTCOMES** | | *(based on ESC-2021)* | | *(based on NHG-2024)* | | *≤0.9 (PAD) ^(b)^* | | *≥1.4 ^(b)^* | | *(cfPWV; continuous)* | |
|  |  | *RR* | *p* | *OR* | *p* | *OR* | *p* | *OR* | *p* | *β* | *p* |
|  |  |  |  |  |  |  |  |  |  |  |  |
| Per 60 minute decrease | Model 1 ^(c)^ | 1.00 [0.87-1.13] | *0.953* | 1.12 [0.88-1.43] | *0.362* | 1.42 [1.02-1.98] | *0.037* | 1.09 [0.69-1.71] | *0.707* | -0.07 [-0.25-0.11] | *0.437* |
|  | Model 2 ^(d)^ | 1.03 [0.90-1.17] | *0.677* | 1.17 [0.91-1.51] | *0.231* | 1.41 [1.01-1.99] | *0.047* | 1.12 [0.70-1.78] | *0.642* | -0.07 [-0.26-0.11] | *0.422* |
|  | Model 3 ^(e)^ | 1.02 [0.90-1.17] | *0.731* | 1.15 [0.89-1.49] | *0.272* | 1.46 [0.96-2.21] | *0.074* | 1.11 [0.69-1.77] | *0.667* | -0.08 [-0.26-0.10] | *0.407* |
|  |  |  |  |  |  |  |  |  |  |  |  |
| Tertile 1 (low)  ^(a)^ | Model 1 ^(c)^ | 0.94 [0.71-1.26] | *0.680* | 1.10 [0.62-1.94] | *0.752* | 2.82 [1.22-6.53] | *0.016* | 1.52 [0.62-3.71] | *0.359* | -0.13 [-0.52-0.27] | *0.538* |
|  | Model 2 ^(d)^ | 1.04 [0.78-1.40] | *0.785* | 1.26 [0.70-2.27] | *0.440* | 2.87 [1.21-6.78] | *0.016* | 1.51 [0.60-3.76] | *0.380* | -0.12 [-0.52-0.28] | *0.566* |
|  | Model 3 ^(e)^ | 1.03 [0.77-1.38] | *0.842* | 1.23 [0.68-2.22] | *0.498* | 3.43 [1.22-9.65] | *0.019* | 1.50 [0.60-3.78] | *0.389* | -0.12 [-0.52-0.28] | *0.546* |
|  |  |  |  |  |  |  |  |  |  |  |  |
| Tertile 2 (medium)  ^(a)^ | Model 1 ^(c)^ | 0.91 [0.68-1.22] | *0.520* | 1.43 [0.82-2.48] | *0.203* | 2.03 [0.85-4.87] | *0.112* | 0.34 [0.09-1.27] | *0.109* | -0.20 [-0.60-0.21] | *0.337* |
|  | Model 2 ^(d)^ | 0.94 [0.70-1.26] | *0.675* | 1.48 [0.84-2.60] | *0.177* | 1.90 [0.78-4.61] | *0.157* | 0.34 [0.09-1.31] | *0.116* | -0.20 [-0.60-0.20] | *0.337* |
|  | Model 3 ^(e)^ | 0.94 [0.70-1.26] | *0.681* | 1.43 [0.81-2.52] | *0.223* | 1.65 [0.59-4.64] | *0.341* | 0.32 [0.08-1.23] | *0.097* | -0.16 [-0.57-0.24] | *0.422* |
|  |  |  |  |  |  |  |  |  |  |  |  |

*T_50_ was operationalized per 60-minute decrease and as categorical variable. HFpEF was based on ESC-2021 and NHG-2024 guidelines. The ABI was categorized as ≤0.9 (PAD), 0.9-1.4 (normal, reference category) and ≥1.4 (high). Central arterial stiffness was continuously measured per 1 m/s increment. Values within square brackets are the 95%-C.I..*

*cfPWV = Carotid-femoral Pulse Wave Velocity, C.I. = Confidence Interval, CVD = Cardiovascular Disease, ESC = European Society of Cardiology, HFpEF = Heart Failure with preserved Ejection Fraction, NHG = Dutch Primary Care, OR = Odds Ratio, PAD = Peripheral Artery Disease, RR = Risk Ratio.*

1. *The reference is T_50_ tertile 3 (high).*
2. *The reference category is 0.9<ABI<1.4.*

*(c) Adjusted for age (years), sex (male/female) and duration of diabetes (<14 years / ≥14 years).*

*(d) Adjusted for model 1 + BMI (<30 kg/m^2^ / ≥30 kg/m^2^), hypertension (yes/no), hypercholesterolemia (yes/no), smoking status (ever/never), alcohol intake (non-drinker/drinker) and eGFR (<90 mL/min/1.73m^2^ / ≥90 mL/min/1.73m^2^).*

*(e) Adjusted for model 2 + insulin drug use (yes/no), history of CVD (yes/no) and HbA1c (<52 mmol/mol / 52-62 mmol/mol / >62 mmol/mol).*

**Table S2:** *Associations between T_50_ with HFpEF, the ankle-brachial index and central arterial stiffness.*

**Table S3:** *Associations between T_50_ with peripheral arterial calcification score and coronary arterial calcification score.*

| **CALCIFICATION SCORE** | | | | | | | *Tertile 1 (low) ^(b)^* | | | | | *Tertile 2 (medium) ^(b)^* | | | | | | | | *Tertile 3 (high) ^(b)^* | | | | | |  |
| --- | --- | --- | --- | --- | --- | --- | --- | --- | --- | --- | --- | --- | --- | --- | --- | --- | --- | --- | --- | --- | --- | --- | --- | --- | --- | --- |
|  | |  |  | | | *OR* | | | | *p* | | | | *OR* | | | *p* | | | *OR* | | | *p* | | |  |
|  | |  |  | | |  | | | |  | | | |  | | |  | | |  | | |  | | |  |
| **Peripheral Calcification Score** | Per 60 minute decrease | | Model 1 ^(c)^ | | 1.11 [0.87-1.42] | | | | *0.407* | | | | 1.23 [0.95-1.59] | | | *0.109* | | | 1.33 [1.02-1.74] | | | *0.035* | | |  |  |
|  |  |  | Model 2 ^(d)^ | | 1.06 [0.83-1.37] | | | | *0.624* | | | | 1.22 [0.93-1.58] | | | *0.145* | | | 1.32 [1.00-1.73] | | | *0.049* | | |  |  |
|  |  |  | Model 3 ^(e)^ | | 1.07 [0.83-1.37] | | | | *0.611* | | | | 1.20 [0.92-1.57] | | | *0.172* | | | 1.28 [0.96-1.69] | | | *0.088* | | |  |  |
|  |  | |  | |  | | | |  | | | |  | | |  | | |  | | |  | | |  |  |
|  | Tertile 1 (low)  ^(a)^ | | Model 1 ^(c)^ | | 1.20 [0.68-2.13] | | | | *0.526* | | | | 1.85 [1.02-3.36] | | | *0.042* | | | 1.56 [0.84-2.90] | | | *0.158* | | |  |  |
|  |  |  | Model 2 ^(d)^ | | 1.11 [0.62-1.99] | | | | *0.726* | | | | 1.89 [1.03-3.48] | | | *0.040* | | | 1.60 [0.85-3.02] | | | *0.147* | | |  |  |
|  |  |  | Model 3 ^(e)^ | | 1.12 [0.62-2.01] | | | | *0.716* | | | | 1.85 [1.00-3.42] | | | *0.050* | | | 1.51 [0.79-2.89] | | | *0.211* | | |  |  |
|  |  | |  | |  | | | |  | | | |  | | |  | | |  | | |  | | |  |  |
|  | Tertile 2 (medium)  ^(a)^ | | Model 1 ^(c)^ | | 0.59 [0.34-1.02] | | | | *0.060* | | | | 0.66 [0.37-1.19] | | | *0.168* | | | 0.62 [0.34-1.14] | | | *0.124* | | |  |  |
|  |  |  | Model 2 ^(d)^ | | 0.59 [0.34-1.04] | | | | *0.069* | | | | 0.66 [0.37-1.20] | | | *0.171* | | | 0.63 [0.34-1.16] | | | *0.135* | | |  |  |
|  |  |  | Model 3 ^(e)^ | | 0.61 [0.34-1.06] | | | | *0.081* | | | | 0.63 [0.35-1.14] | | | *0.128* | | | 0.55 [0.29-1.03] | | | *0.062* | | |  |  |
|  |  | |  | |  | | | |  | | | |  | | |  | | |  | | |  | | |  |  |
| **Coronary Calcification Score** | Per 60 minute decrease | | Model 1 ^(c)^ | | 1.36 [1.00-1.85] | | | | *0.048* | | | | 1.50 [1.09-2.06] | | | *0.012* | | | 1.69 [1.22-2.33] | | | *0.002* | | |  |  |
|  |  |  | Model 2 ^(d)^ | | 1.34 [0.98-1.84] | | | | *0.063* | | | | 1.50 [1.08-2.07] | | | *0.014* | | | 1.67 [1.20-2.33] | | | *0.003* | | |  |  |
|  |  |  | Model 3 ^(e)^ | | 1.36 [0.99-1.87] | | | | *0.054* | | | | 1.51 [1.09-2.10] | | | *0.013* | | | 1.62 [1.15-2.29] | | | *0.006* | | |  |  |
|  |  | |  | |  | | | |  | | | |  | | |  | | |  | | |  | | |  |  |
|  | Tertile 1 (low)  ^(a)^ | | Model 1 ^(c)^ | | 1.81 [0.92-3.57] | | | | *0.087* | | | | 1.98 [0.99-3.96] | | | *0.055* | | | 2.67 [1.30-5.47] | | | *0.008* | | |  |  |
|  |  |  | Model 2 ^(d)^ | | 1.73 [0.87-3.46] | | | | *0.119* | | | | 2.04 [1.00-4.14] | | | *0.050* | | | 2.70 [1.30-5.64] | | | *0.008* | | |  |  |
|  |  |  | Model 3 ^(e)^ | | 1.75 [0.88-3.51] | | | | *0.112* | | | | 2.03 [0.99-4.15] | | | *0.052* | | | 2.56 [1.20-5.47] | | | *0.015* | | |  |  |
|  |  | |  | |  | | | |  | | | |  | | |  | | |  | | |  | | |  |  |
|  | Tertile 2 (medium)  ^(a)^ | | Model 1 ^(c)^ | | 1.80 [0.91-3.55] | | | | *0.093* | | | | 1.29 [0.64-2.63] | | | *0.477* | | | 1.88 [0.91-3.89] | | | *0.089* | | |  |  |
|  |  |  | Model 2 ^(d)^ | | 1.83 [0.92-3.66] | | | | *0.086* | | | | 1.34 [0.65-2.75] | | | *0.428* | | | 1.92 [0.92-4.01] | | | *0.084* | | |  |  |
|  |  |  | Model 3 ^(e)^ | | 1.84 [0.92-3.68] | | | | *0.086* | | | | 1.29 [0.62-2.67] | | | *0.490* | | | 1.59 [0.74-3.44] | | | *0.237* | | |  |  |
|  | |  | |  | | | |  | | |  | | | |  | | |  | | |  | | |  | | |

*T_50_ was operationalized per 60-minute decrease and as categorical variable. Peripheral arterial calcification score and coronary arterial calcification score were both operationalized into zero + tertiles>0. Values within square brackets are the 95%-C.I..*

*C.I. = Confidence Interval, OR = Odds Ratio.*

1. *The reference is T_50_ tertile 3 (high).*
2. *The reference is no calcification (score = 0).*

*(c) Adjusted for age (years), sex (male/female) and duration of diabetes (<14 years / ≥14 years).*

*(d) Adjusted for model 1 + BMI (<30 kg/m^2^ / ≥30 kg/m^2^), hypertension (yes/no), hypercholesterolemia (yes/no), smoking status (ever/never), alcohol intake (non-drinker/drinker) and eGFR (<90 mL/min/1.73m^2^ / ≥90 mL/min/1.73m^2^).*

*(e) Adjusted for model 2 + insulin drug use (yes/no), history of CVD (yes/no) and HbA1c (<52 mmol/mol / 52-62 mmol/mol / >62 mmol/mol).*

*E. Sensitivity Analysis with complete case analysis*

HFpEF (ESC-2021) n = 172

HFpEF (NHG-2024) n = 172

ABI n = 178

Arterial Stiffness n = 165

Calcification n = 167

|  |  | **HFpEF** | | **HFpEF** | | **Ankle-Brachial Index** | | | | **Arterial Stiffness** | |
| --- | --- | --- | --- | --- | --- | --- | --- | --- | --- | --- | --- |
| **CVD OUTCOMES** | | *(based on ESC-2021)* | | *(based on NHG-2024)* | | *≤0.9 (PAD) ^(b)^* | | *≥1.4 ^(b)^* | | *(cfPWV; continuous)* | |
|  |  | *RR* | *p* | *OR* | *p* | *OR* | *p* | *OR* | *p* | *β* | *p* |
|  |  |  |  |  |  |  |  |  |  |  |  |
| Per 60 minute decrease | Model 1 ^(c)^ | 1.00 [0.79-1.26] | *0.991* | 1.02 [0.65-1.60] | *0.914* | 1.76 [0.94-3.27] | *0.075* | 1.56 [0.68-3.55] | *0.294* | 0.05 [-0.35-0.44] | *0.819* |
|  | Model 2 ^(d)^ | 0.98 [0.76-1.24] | *0.845* | 1.00 [0.62-1.60] | *0.991* | 1.59 [0.87-2.92] | *0.133* | 1.80 [0.61-5.36] | *0.289* | 0.08 [-0.32-0.48] | *0.704* |
|  | Model 3 ^(e)^ | 0.92 [0.70-1.19] | *0.517* | 0.94 [0.53-1.61] | *0.813* | 2.13 [0.63-7.21] | *0.222* | 1.99 [0.64-6.18] | *0.233* | 0.28 [-0.14-0.70] | *0.194* |
|  |  |  |  |  |  |  |  |  |  |  |  |
| ≤330 minutes  ^(a)^ | Model 1 ^(c)^ | 1.14 [0.63-2.09] | *0.673* | 0.81 [0.26-2.60] | *0.725* | 3.11 [0.60-16.07] | *0.175* | 4.19 [0.47-37.47] | *0.200* | 0.19 [-0.72-1.09] | *0.686* |
|  | Model 2 ^(d)^ | 1.13 [0.62-2.08] | *0.690* | 0.87 [0.26-2.90] | *0.815* | 3.18 [0.60-17.00] | *0.176* | 3.27 [0.32-33.20] | *0.317* | 0.19 [-0.73-1.11] | *0.686* |
|  | Model 3 ^(e)^ | 1.00 [0.54-1.87] | *0.988* | 0.75 [0.20-2.81] | *0.659* | 24.98 [0.69-908.13] | *0.079* | 3.98 [0.37-43.25] | *0.257* | 0.60 [-0.35-1.55] | *0.213* |
|  |  |  |  |  |  |  |  |  |  |  |  |
| 330-390 minutes  ^(a)^ | Model 1 ^(c)^ | 1.11 [0.61-2.06] | *0.726* | 1.45 [0.51-4.32] | *0.488* | 1.39 [0.21-9.07] | *0.731* | No estimation |  | -0.71 [-1.64-0.21] | *0.129* |
|  | Model 2 ^(d)^ | 1.15 [0.62-2.15] | *0.659* | 1.72 [0.57-5.49] | *0.341* | 1.25 [0.18-8.46] | *0.822* | No estimation |  | -0.72 [-1.66-0.22] | *0.134* |
|  | Model 3 ^(e)^ | 1.03 [0.54-2.00] | *0.934* | 1.38 [0.39-5.25] | *0.621* | 5.15 [0.09-288.92] | *0.425* | No estimation |  | -0.45 [-1.45-0.55] | *0.379* |
|  |  |  |  |  |  |  |  |  |  |  |  |

*T_50_ was operationalized per 60-minute decrease and as categorical variable. HFpEF was based on ESC-2021 and NHG-2024 guidelines. The ABI was categorized as ≤0.9 (PAD), 0.9-1.4 (normal, reference category) and ≥1.4 (high). Central arterial stiffness was continuously measured per 1 m/s increment. Values within square brackets are the 95%-C.I..*

*cfPWV = Carotid-femoral Pulse Wave Velocity, C.I. = Confidence Interval, CVD = Cardiovascular Disease, ESC = European Society of Cardiology, HFpEF = Heart Failure with preserved Ejection Fraction, NHG = Dutch Primary Care, OR = Odds Ratio, PAD = Peripheral Artery Disease, RR = Risk Ratio.*

1. *The reference is T_50_≥390 minutes.*
2. *The reference category is 0.9<ABI<1.4.*

*(c) Adjusted for age (years), sex (male/female) and duration of diabetes (<14 years / ≥14 years).*

*(d) Adjusted for model 1 + BMI (<30 kg/m^2^ / ≥30 kg/m^2^), hypertension (yes/no), hypercholesterolemia (yes/no), smoking status (ever/never), alcohol intake (non-drinker/drinker) and eGFR (<90 mL/min/1.73m^2^ / ≥90 mL/min/1.73m^2^).*

*(e) Adjusted for model 2 + insulin drug use (yes/no), history of CVD (yes/no) and HbA1c (<52 mmol/mol / 52-62 mmol/mol / >62 mmol/mol).*

**Table S4:** *Associations between T_50_ with HFpEF, the ankle-brachial index and central arterial stiffness.*

**Table S5:** *Associations between T_50_ with peripheral arterial calcification score and coronary arterial calcification score.*

| **CALCIFICATION SCORE** | | | | | | | *Tertile 1 (low) ^(b)^* | | | | | *Tertile 2 (medium) ^(b)^* | | | | | | | | *Tertile 3 (high) ^(b)^* | | | | | |  |
| --- | --- | --- | --- | --- | --- | --- | --- | --- | --- | --- | --- | --- | --- | --- | --- | --- | --- | --- | --- | --- | --- | --- | --- | --- | --- | --- |
|  | |  |  | | | *OR* | | | | *p* | | | | *OR* | | | *p* | | | *OR* | | | *p* | | |  |
|  | |  |  | | |  | | | |  | | | |  | | |  | | |  | | |  | | |  |
| **Peripheral Calcification Score** | Per 60 minute decrease | | Model 1 ^(c)^ | | 0.89 [0.55-1.44] | | | | *0.644* | | | | 1.00 [0.61-1.64] | | | *0.996* | | | 1.13 [0.69-1.85] | | | *0.637* | | |  |  |
|  |  |  | Model 2 ^(d)^ | | 0.87 [0.53-1.45] | | | | *0.599* | | | | 0.93 [0.56-1.56] | | | *0.788* | | | 1.09 [0.64-1.85] | | | *0.761* | | |  |  |
|  |  |  | Model 3 ^(e)^ | | 0.72 [0.39-1.32] | | | | *0.286* | | | | 0.74 [0.41-1.35] | | | *0.326* | | | 0.95 [0.53-1.73] | | | *0.878* | | |  |  |
|  |  | |  | |  | | | |  | | | |  | | |  | | |  | | |  | | |  |  |
|  | ≤330 minutes ^(a)^ | | Model 1 ^(c)^ | | 0.79 [0.22-2.76] | | | | *0.708* | | | | 0.87 [0.26-2.94] | | | *0.819* | | | 0.85 [0.24-3.03] | | | *0.808* | | |  |  |
|  |  |  | Model 2 ^(d)^ | | 0.67 [0.18-2.45] | | | | *0.541* | | | | 0.76 [0.21-2.69] | | | *0.665* | | | 0.74 [0.20-2.78] | | | *0.658* | | |  |  |
|  |  |  | Model 3 ^(e)^ | | 0.48 [0.11-2.13] | | | | *0.332* | | | | 0.46 [0.11-1.93] | | | *0.288* | | | 0.53 [0.12-2.30] | | | *0.399* | | |  |  |
|  |  | |  | |  | | | |  | | | |  | | |  | | |  | | |  | | |  |  |
|  | 330-390 minutes ^(a)^ | | Model 1 ^(c)^ | | 0.97 [0.30-3.13] | | | | *0.960* | | | | 0.50 [0.14-1.74] | | | *0.275* | | | 0.71 [0.20-2.53] | | | *0.596* | | |  |  |
|  |  |  | Model 2 ^(d)^ | | 0.92 [0.28-3.07] | | | | *0.894* | | | | 0.50 [0.14-1.86] | | | *0.303* | | | 0.67 [0.18-2.53] | | | *0.551* | | |  |  |
|  |  |  | Model 3 ^(e)^ | | 0.73 [0.18-3.04] | | | | *0.666* | | | | 0.28 [0.06-1.33] | | | *0.110* | | | 0.47 [0.10-2.12] | | | *0.326* | | |  |  |
|  |  | |  | |  | | | |  | | | |  | | |  | | |  | | |  | | |  |  |
| **Coronary Calcification Score** | Per 60 minute decrease | | Model 1 ^(c)^ | | 1.58 [0.84-2.95] | | | | *0.156* | | | | 1.94 [1.01-3.70] | | | *0.046* | | | 1.80 [0.94-3.44] | | | *0.074* | | |  |  |
|  |  |  | Model 2 ^(d)^ | | 1.64 [0.83-3.22] | | | | *0.153* | | | | 2.01 [1.00-4.06] | | | *0.051* | | | 1.85 [0.92-3.72] | | | *0.082* | | |  |  |
|  |  |  | Model 3 ^(e)^ | | 1.59 [0.71-3.58] | | | | *0.262* | | | | 2.16 [0.94-4.98] | | | *0.070* | | | 1.80 [0.77-4.18] | | | *0.172* | | |  |  |
|  |  | |  | |  | | | |  | | | |  | | |  | | |  | | |  | | |  |  |
|  | ≤330 minutes ^(a)^ | | Model 1 ^(c)^ | | 3.71 [0.65-21.28] | | | | *0.141* | | | | 3.68 [0.62-22.05] | | | *0.153* | | | 3.97 [0.66-23.81] | | | *0.131* | | |  |  |
|  |  |  | Model 2 ^(d)^ | | 3.26 [0.55-19.34] | | | | *0.193* | | | | 3.66 [0.58-22.96] | | | *0.165* | | | 3.80 [0.61-23.86] | | | *0.154* | | |  |  |
|  |  |  | Model 3 ^(e)^ | | 2.76 [0.39-19.37] | | | | *0.307* | | | | 3.44 [0.46-25.53] | | | *0.227* | | | 3.09 [0.39-24.30] | | | *0.264* | | |  |  |
|  |  | |  | |  | | | |  | | | |  | | |  | | |  | | |  | | |  |  |
|  | 330-390 minutes ^(a)^ | | Model 1 ^(c)^ | | 0.80 [0.22-2.91] | | | | *0.735* | | | | 1.00 [0.26-3.80] | | | *0.996* | | | 0.89 [0.23-3.48] | | | *0.868* | | |  |  |
|  |  |  | Model 2 ^(d)^ | | 0.75 [0.20-2.81] | | | | *0.668* | | | | 1.16 [0.29-4.65] | | | *0.838* | | | 0.90 [0.22-3.75] | | | *0.890* | | |  |  |
|  |  |  | Model 3 ^(e)^ | | 0.72 [0.15-3.46] | | | | *0.685* | | | | 0.91 [0.17-4.72] | | | *0.908* | | | 0.68 [0.12-3.93] | | | *0.666* | | |  |  |
|  | |  | |  | | | |  | | |  | | | |  | | |  | | |  | | |  | | |

*T_50_ was operationalized per 60-minute decrease and as categorical variable. Peripheral arterial calcification score and coronary arterial calcification score were both operationalized into zero + tertiles>0. Values within square brackets are the 95%-C.I..*

*C.I. = Confidence Interval, OR = Odds Ratio.*

1. *The reference is T_50_≥390 minutes.*
2. *The reference is no calcification (score = 0).*

*(c) Adjusted for age (years), sex (male/female) and duration of diabetes (<14 years / ≥14 years).*

*(d) Adjusted for model 1 + BMI (<30 kg/m^2^ / ≥30 kg/m^2^), hypertension (yes/no), hypercholesterolemia (yes/no), smoking status (ever/never), alcohol intake (non-drinker/drinker) and eGFR (<90 mL/min/1.73m^2^ / ≥90 mL/min/1.73m^2^).*

*(e) Adjusted for model 2 + insulin drug use (yes/no), history of CVD (yes/no) and HbA1c (<52 mmol/mol / 52-62 mmol/mol / >62 mmol/mol).*
